# Expression profiling and integrative analysis of the *CESA*/*CSL *superfamily in rice

**DOI:** 10.1186/1471-2229-10-282

**Published:** 2010-12-20

**Authors:** Lingqiang Wang, Kai Guo, Yu Li, Yuanyuan Tu, Huizhen Hu, Bingrui Wang, Xiaocan Cui, Liangcai Peng

**Affiliations:** 1 National Key Laboratory of Crop Genetic Improvement, Biomass and Bioenergy Research Centre, Huazhong Agricultural University, Wuhan, Hubei, 430070, PR China; 2 College of Plant Sciences and Technology, Huazhong Agricultural University, Wuhan, Hubei, 430070, PR China; 3 College of Life Sciences and Technology, Huazhong Agricultural University, Wuhan, Hubei, 430070, PR China

## Abstract

**Background:**

The cellulose synthase and cellulose synthase-like gene superfamily (*CESA*/*CSL*) is proposed to encode enzymes for cellulose and non-cellulosic matrix polysaccharide synthesis in plants. Although the rice (*Oryza sativa *L.) genome has been sequenced for a few years, the global expression profiling patterns and functions of the *OsCESA*/*CSL *superfamily remain largely unknown.

**Results:**

A total of 45 identified members of *OsCESA*/*CSL *were classified into two clusters based on phylogeny and motif constitution. Duplication events contributed largely to the expansion of this superfamily, with Cluster I and II mainly attributed to tandem and segmental duplication, respectively. With microarray data of 33 tissue samples covering the entire life cycle of rice, fairly high *OsCESA *gene expression and rather variable *OsCSL *expression were observed. While some members from each *CSL *family (*A1*, *C9*, *D2*, *E1*, *F6 *and *H1*) were expressed in all tissues examined, many of *OsCSL *genes were expressed in specific tissues (stamen and radicles). The expression pattern of *OsCESA*/*CSL *and *OsBC1L *which extensively co-expressed with *OsCESA*/*CSL *can be divided into three major groups with ten subgroups, each showing a distinct co-expression in tissues representing typically distinct cell wall constitutions. In particular, *OsCESA1, -3 & -8 *and *OsCESA4, -7 & -9 *were strongly co-expressed in tissues typical of primary and secondary cell walls, suggesting that they form as a cellulose synthase complex; these results are similar to the findings in *Arabidopsis*. *OsCESA5*/*OsCESA6 *is likely partially redundant with *OsCESA3 *for OsCESA complex organization in the specific tissues (plumule and radicle). Moreover, the phylogenetic comparison in rice, *Arabidopsis *and other species can provide clues for the prediction of orthologous gene expression patterns.

**Conclusions:**

The study characterized the *CESA*/*CSL *of rice using an integrated approach comprised of phylogeny, transcriptional profiling and co-expression analyses. These investigations revealed very useful clues on the major roles of *CESA*/*CSL*, their potentially functional complement and their associations for appropriate cell wall synthesis in higher plants.

## Background

Plant cell walls make up the most abundant renewable biomass on the earth. Of the main wall polysaccharides, cellulose is synthesized at the plasma membrane whereas non-cellulosic polysaccharides (pectins and hemicelluloses) are made in the Golgi body. In higher plants, *CESA *was first isolated from developing cotton fibers, and it was further characterized in *Arabidopsis *as catalytic subunits of cellulose synthase complexes (CSCs) that locate within the plasma membrane [1,2]. The CSCs are believed to be a rosette structure holding as many as 36 individual CESA proteins. In *Arabidopsis*, at least three CESA isoforms are required for the synthesis of primary (AtCESA1, -3 & -6) and secondary (AtCESA4, -7 & -8) cell walls. Mutant and co-immunoprecipitation analysis demonstrates that AtCESA2 & -5 are partially redundant with AtCESA6 [3-5]. Consequently, the *CESA *family has been identified in other plants, such as maize [6], barley [7], poplar [8,9], pine [10], moss [11] and rice [12]. Those higher plants appear to have many more *CESA *family members, but very little is known about their functions in comparison to those from *Arabidopsis*.

A large number of cellulose synthase-like (*CSL*) genes showing sequence similarity to *CESA *have been identified. In *Arabidopsis*, a total of 30 *CSL *genes are classified into the six following families: *CSLA*, *B*, *C*, *D*, *E *and *G *[13]. Based on the common motif DXD, D, Q/RXXRW, all CSL proteins are predicted to encode processive glycosyl transferases (GTs) [14-17]. There are increasing lines of evidence supporting CSL as catalytic enzymes for non-cellulosic polysaccharide synthesis. In *Arabidopsis *and guar, genes of the *CSLA *family are demonstrated to encode (1,4)-*β*-D-mannan synthases [16-19]; in rice, genes of the *CSLF *family have been implicated in the biosynthesis of (1,3;1,4)-*β*-D-glucans [20]. More recently, it has also been established that barley *CSLH *genes, like *CSLF*, are able to direct mixed-linkage *β*-glucan biosynthesis [21]. In addition, the *CSLC *family contains a glucan synthase involved in the synthesis of the backbone of xyloglucan [22,23], and several *CSLD *mutants have been characterized for their potential roles in wall polysaccharide (xylan and homogalacturonan) synthesis [24-27]. However, even though there are a number of *CSLD *mutants in *Arabidopsis *and rice displaying interesting phenotypes, very little is known about the biochemical function(s) of CSLD proteins. The detailed functions of these CSL genes, especially those of families CSLB, E and G, remain to be clarified.

Rice, one of the major food crops across the world, is a model species for the functional genomic characterization of monocotyledonous plants. With the completion of the rice genome sequence, the *CESA*/*CSL *superfamily has been identified in rice http://waltonlab.prl.msu.edu/CSL_updates.htm. This rice superfamily has shown a striking difference in the *CSL *families between rice and *Arabidopsis*, reflecting the distinct cell wall compositions of dicots and monocots [28]. In contrast, several orthologs of the *AtCSL *genes exhibited a similar function in rice [29]. But, the *OsCESA*/*CSL *functions still remain largely unknown.

In this work, we utilized an innovative approach for the characterization of genes of the *CESA*/*CSL *superfamily in higher plants. We first performed a phylogenetic and structural analysis to determine their potential functions. Then, we focused on an integrative analysis of co-expression profiling and regulations using 33 tissue samples from the entire life cycle of two rice varieties. We further carried out a comparative analysis of *CESA*/*CSL *in rice and *Arabidopsis*.

## Methods

### Database searches for OsCESA/CSL genes in rice

The Hidden Markov Model (HMM) profile of the cellulose synthase domain (PF03552) was downloaded from PFam http://pfam.sanger.ac.uk/. We employed a name search and the protein family ID PF03552 for the identification of *OsCESA*/*CSL *genes from the rice genome. Information about the chromosomal localization, coding sequence (CDS), amino acid (AA) and full length cDNA accessions was obtained from TIGR http://www.tigr.org and KOME http://cdna01.dna.affrc.go.jp/cDNA. The corresponding protein sequences were confirmed by the Pfam database http://www.sanger.ac.uk/Software/Pfam/search.shtml.

### Sequence and structure analysis

We performed our exon-intron structure analysis using GSDS http://gsds.cbi.pku.edu.cn/[30]. The protein transmembrane helices were predicted by the TMHMM Server V2.0 http://www.cbs.dtu.dk/services/TMHMM/[31,32]. Protein subcellular locations were analyzed using WoLF PSORT http://psort.nibb.ac.jp/[33], an extension of the PSORT II program http://www.psort.org.

### Phylogenetic analyses and motif identification

The multiple alignment analysis was performed using the Clustal X program (version 1.83) [34] and MAFFT [35]. The unrooted phylogenetic trees were constructed with the MEGA3.1 program and the neighbor joining method [36] with 1,000 bootstrap replicates. Protein sequences were analyzed using the MEME program http://meme.sdsc.edu/meme/cgi-bin/meme.cgi for the confirmation of the motifs. The MEME program (version 4.0) was employed with the following parameters: number of repetitions, any; maximum number of motifs, 25; optimum motif width set to >6 and <200. The motifs were annotated using the InterProScan http://www.ebi.ac.uk/Tools/InterProScan/ search program.

### Chromosomal localization and gene duplication

The *OsCESA/CSL *genes were mapped on chromosomes by identifying their chromosomal positions given in the TIGR rice database. The duplicated genes were elucidated from the segmental genome duplication of rice http://www.tigr.org/tdb/e2k1/osa1/segmental_dup/100. The DAGchainer program [37] was used to determine the segmental duplications with following parameters: V = 5 B = 5 E = 1e-10-filter seg and distance = 100 kb. Genes separated by five or fewer genes were considered to be tandem duplicates. The distance between these genes on the chromosomes was calculated, and the percentage of protein sequence similarity was determined by the MegAlign software 4.0.

### Genome-wide expression analysis of OsCESA/CSL and OsBC1L in rice and AtCESA/CSL and AtCOBL in Arabidopsis

The expression profile data of *OsCESA*/*CSL *in 33 tissue examples (Additional file 1) of Zhenshan 97 (ZS97) and Minghui 63 (MH63) were obtained from the CREP database http://crep.ncpgr.cn and from a rice transcriptome project using the Affymetrix Rice GeneChip microarray (Additional file 2). Massively parallel signature sequencing (MPSS) data http://mpss.udel.edu/rice/ was used to determine the expression profiles of the genes with conflicting probe set signals. The expression values were log-transformed, and cluster analyses were performed using a software cluster with Euclidean distances and the hierarchical cluster method of "complete linkage clustering". The clustering tree was constructed and viewed in Java Treeview. The same method was used in the "artificial mutant" analysis. However, in the hierarchical cluster of the "artificial mutant" analysis, the expression data for regarding gene(s) or tissues were deleted. All *Arabidopsis *microarray data were downloaded from the Gene Expression Omnibus database http://www.ncbi.nlm.nih.gov/geo/ using the GSE series accession numbers GSE5629, GSE5630, GSE5631, GSE5632, GSE5633 and GSE5634 (Additional file 3 and 4). Subsequent analysis of the gene expression data was performed in the statistical computing language R http://www.r-project.org using packages available from the Bioconductor project http://www.bioconductor.org. The raw data were processed with the Affymetrix Microarray Analysis Suite (MAS Version 5, Affymetrix) [38].

### RT-PCR analysis of representative genes of the OsCESA/CSLD family

The primers designed for the RT-PCR analysis are listed in Additional file 5. Samples were collected from Zhenshan 97 (ZS97), one of the varieties used in microarray. The samples were ground in liquid nitrogen using a mortar and pestle. Total RNA (4 μg) was isolated using a RNA extraction kit (TransZol reagent, TransGen) and treated with RNase-free DNase I (Invitrogen) for 15 min to eliminate possible contaminating DNA. Then, first strand cDNA was reverse transcribed from total RNA with an oligo(dT)_18 _primer in a 50 μl reaction (diluted to 200 μl before use) using an M-MLV Reverse Transcriptase (Promega) according to the manufacturer's instructions. For the PCR amplification of the reverse transcription product, the PCR reaction was performed in a volume of 25 μl containing 2 μl of template. The reactions were conducted with rTaq polymerase (Takara Biotechnology, Japan) on a Bio-rad MyCycler thermal cycler using the following program: 3 min at 95°C for pre-denaturation, followed by 29 cycles of 20 s at 95°C, 20 s at 60°C and 30 s at 72°C, and a final 5 min extension at 72°C.

### Plant cell wall fractionation and polysaccharide colorimetric assays

The plant tissues were firstly heated at 110-120°C for about 10 min to inactivate the enzymes, before they were fully ground in a mortar and pestle with liquid nitrogen and dried to constant weight at 65°C for about 2 days. The extraction and fractionation of the cell wall polysaccharides were performed with 0.5 M phosphate buffer, chloroform-methanol (1:1, V/V), DMSO-water (9:1, V/V), 0.5% ammonium oxalate, 4 M KOH, acetic acid-nitric acid-water (8:1:2, V/V/V) and 72% (w/w) H_2_SO_4_, and the extraction was measured using colorimetric assays according the method reported in a previous study [39].

## Results

### OsCESA/CSL superfamily in rice

Searching the TIGR database revealed 45 sequences that significantly matched to *CESA/CSL *superfamily, out of which eleven are predicted as *OsCESA *and 34 as *OsCSL *http://waltonlab.prl.msu.edu/CSL_updates.htm (Table 1). The sequences of *OsCESA10 *were short and appeared to be truncated. Of the 11 OsCESA sequences, CESA 1-9 contained a cellulose synthase domain (CS) and zinc finger structure, whereas CESA 10 & -11 only harbored a CS domain. When referring to the CSL classification in *Arabidopsis*, the 34 OsCSL proteins with a CS domain could be divided into six groups (Table 1). In addition, 31 genes had KOME cDNA support, and probes for 41 genes could be found in the CREP database (Table 1). The "DXD, D, QXXRW" motif is typically in the OsCESA/CSL family, but OsCSLA10 and OsCSLE2 showed alternative motifs ("DXD, D, RXXRW" and "DXD, D, LXXRW"); OsCESA10, 11 and CSLH3 contained only "DXD" and lacked "D, LXXRW" (Additional file 6). Besides the "DXD, D, LXXRW" motif, some novel conserved amino acid residues (G, E, G, P and G) with unknown biochemical functions were also detected in this region.

**Table 1:** List of the 45 OsCESA/CSL genes identified in rice

No.	Genes	Accession Number		Probsets^a^	Protein characteristics	
				
		TIGR Loci	KOME cDNA		**Pred Hel**^**b**^	**Domains**^**c**^
1	*OsCESA1*	LOC_Os05g08370	AK100188	Os.10183.1.S2_at	8	Zinc finger, CS (PF03552)
2	*OsCESA2*	LOC_Os03g59340	AK069196	Os.14979.1.S1_at	6	Zinc finger, CS (PF03552)
3	*OsCESA3*	LOC_Os07g24190	AK073561	Os.10178.2.S1_a_at	8	Zinc finger, CS (PF03552)
4	*OsCESA4*	LOC_Os01g54620	AK100475	Os.18724.2.S1_x_at	8	Zinc finger, CS (PF03552)
5	*OsCESA5*	LOC_Os03g62090	AK100877	Os.4857.1.S1_at	8	Zinc finger, CS (PF03552)
6	*OsCESA6*	LOC_Os07g14850	AK100914	Os.10926.1.S1_at	8	Zinc finger, CS (PF03552)
7	*OsCESA7*	LOC_Os10g32980	AK072259	Os.3206.1.S1_at	6	Zinc finger, CS (PF03552)
8	*OsCESA8*	LOC_Os07g10770	AK072356	Os.10176.1.S1_at	6	Zinc finger, CS (PF03552)
9	*OsCESA9*	LOC_Os09g25490	AK121170	Os.10206.1.S1_at	6	Zinc finger, CS (PF03552)
10	*OsCESA10*	LOC_Os12g29300	NF	/	0	CS(PF03552)
11	*OsCESA11*	LOC_Os06g39970	NF	OsAffx.15853.1.S1_at	6	CS(PF03552)
12	*OsCSLA1*	LOC_Os02g09930	AK102694	Os.24972.1.S1_at	5	GT family 2 (PF00535)
13	*OsCSLA2*	LOC_Os10g26630	NF	Os.15231.1.S1_at	5	GT family 2 (PF00535)
14	*OsCSLA3*	LOC_Os06g12460	NF	OsAffx.15389.1.S1_at	5	GT family 2 (PF00535)
15	*OsCSLA4*	LOC_Os03g07350	NF	OsAffx.12764.2.S1_x_at	5	GT family 2 (PF00535)
16	*OsCSLA5*	LOC_Os03g26044	AK111424	Os.56873.1.S1_at	6	GT family 2 (PF00535)
17	*OsCSLA6*	LOC_Os02g51060	AK058756	Os.6170.1.S1_at	5	GT family 2 (PF00535)
18	*OsCSLA7*	LOC_Os07g43710	AK122106	Os.8080.1.S1_at; Os.8080.2.S1_x_at	6	GT family 2 (PF00535)
19	*OsCSLA9*	LOC_Os06g42020	AK242831	Os.48268.1.S1_at	5	GT family 2 (PF00535)
20	*OsCSLA11*	LOC_Os08g33740	NF	OsAffx.6015.1.S1_at	5	GT family 2 (PF00535)
21	*OsCSLC1*	LOC_Os01g56130	AK110759	Os.29016.1.S1_at	5	GT family 2 (PF00535)
22	*OsCSLC2*	LOC_Os09g25900	NF	Os.18770.1.S1_at	4	GT family 2 (PF00535)
23	*OsCSLC3*	LOC_Os08g15420	AK108045	Os.55417.1.S1_at	4	GT family 2 (PF00535)
24	*OsCSLC7*	LOC_Os05g43530	AK243206	Os.15705.1.S1_x_at	2	GT family 2 (PF00535)
25	*OsCSLC9*	LOC_Os03g56060	AK121805	Os.10855.1.S1_at	3	GT family 2 (PF00535)
26	*OsCSLC10*	LOC_Os07g03260	NF	OsAffx.28245.1.S1_at	2	GT family 2 (PF00535)
27	*OsCSLD1*	LOC_Os10g42750	AK110534	Os.46811.1.S1_at	8	CS (PF03552)
28	*OsCSLD2*	LOC_Os06g02180	AK105393	Os.25614.1.S1_at	6	CS (PF03552)
29	*OsCSLD3*	LOC_Os08g25710	NF	OsAffx.17155.1.S1_x_at	6	CS (PF03552)
30	*OsCSLD4*	LOC_Os12g36890	AK242601	Os.57510.1.S1_x_at; Os.57510.1.A1_at	6	CS (PF03552)
31	*OsCSLD5*	LOC_Os06g22980	AK072260	Os.53359.1.S1_at	8	CS (PF03552)
32	*OsCSLE1*	LOC_Os09g30120	AK102766	Os.6165.1.S1_a_at	5	CS (PF03552)
33	*OsCSLE2*	LOC_Os02g49332	AK101487	Os.20406.3.S1_x_at; Os.20406.1.S1_a_at	7	CS (PF03552)
34	*OsCSLE6*	LOC_Os09g30130	AK068464	/	8	CS (PF03552)
35	*OsCSLF1*	LOC_Os07g36700	NF	/	8	CS (PF03552)
36	*OsCSLF2*	LOC_Os07g36690	AK100523	Os.15704.1.S1_at	8	CS (PF03552)
37	*OsCSLF3*	LOC_Os07g36750	NF	OsAffx.5550.1.S1_at	8	CS (PF03552)
38	*OsCSLF4*	LOC_Os07g36740	NF	/	7	CS (PF03552)
39	*OsCSLF6*	LOC_Os08g06380	AK065259	Os.9709.1.A1_at; Os.9709.2.S1_at	9	CS (PF03552)
40	*OsCSLF7*	LOC_Os10g20260	AK110467	Os.46814.1.S1_at	7	CS (PF03552)
41	*OsCSLF8*	LOC_Os07g36630	AK067424	Os.52482.1.S1_at	8	CS (PF03552)
42	*OsCSLF9*	LOC_Os07g36610	AK242890	OsAffx.16586.1.S1_x_at	8	CS (PF03552)
43	*OsCSLH1*	LOC_Os10g20090	AK069071	Os.11623.1.S1_a_at	6	CS (PF03552)
44	*OsCSLH2*	LOC_Os04g35020	NF	Os.45970.1.S1_at	8	CS (PF03552)
45	*OsCSLH3*	LOC_Os04g35030	NF	Os.26822.1.S1_at	2	CS (PF03552)

a Probeset ID of *OsCESA/CSL *genes

b The number of transmembrane helices predicted by the TMHMM server V2.0

c CS, cellulose synthase; GT, glycosyl transferase

### Structural and phylogenetic analyses of OsCESA/CSL

**Figure 1:**
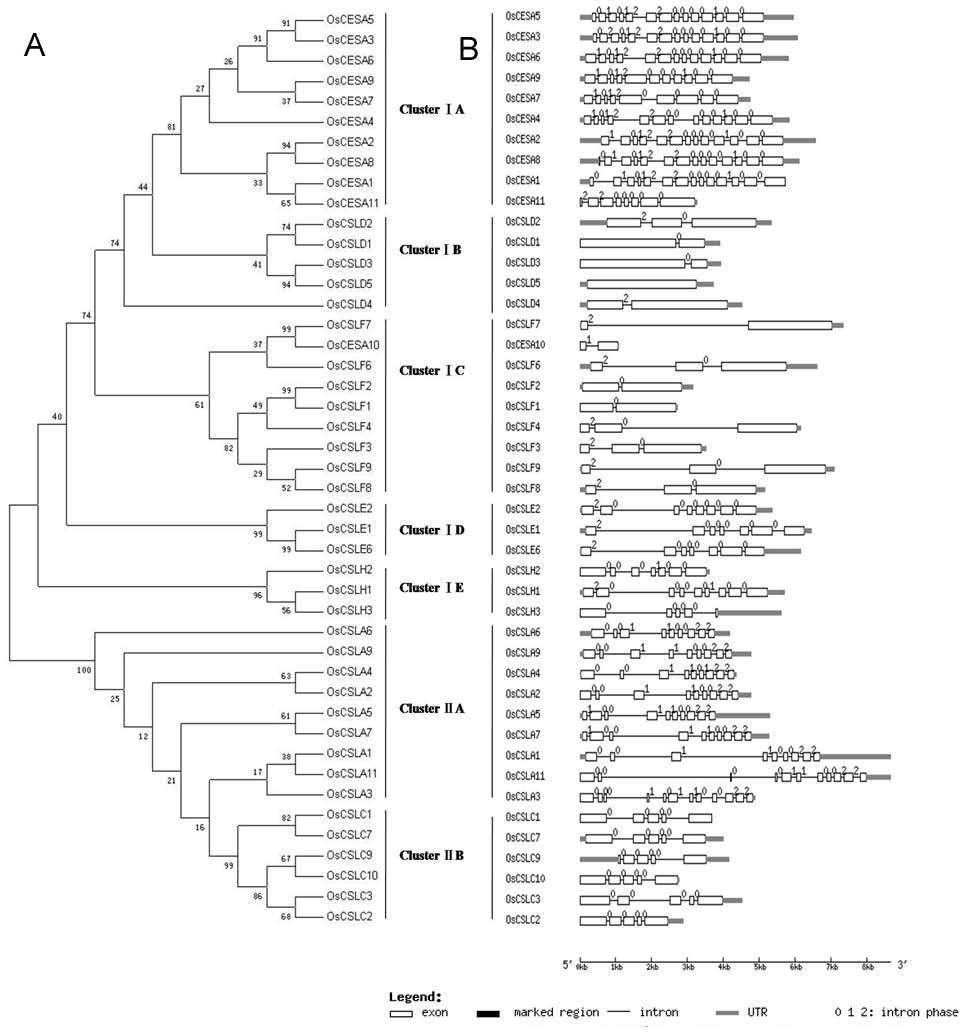
**Unrooted tree of OsCESA/CSL protein family (A) and organization of exons and introns of the corresponding genes (B)**.

An unrooted phylogenetic tree was generated from the alignments of 45 OsCESA/CSL protein sequences with two distinct clusters (Figure 1). Cluster I was resolved into five branches, namely Cluster IA (OsCESA), Cluster IB (OsCSLD), Cluster IC (OsCSLF), Cluster ID (OsCSLE) and Cluster IE (OsCSLH), whereas Cluster II had two branches, Cluster IIA (OsCSLA) and Cluster IIB (OsCSLC). In Cluster I, *OsCESA *had the most introns, and the *OsCSLD *had the fewest number of introns. In Cluster II, *OsCSLA *had more introns than *OsCSLC*. The analysis of motif composition was in agreement with the above OsCESA/CSL family classification (Additional files 7 and 8). Of the total 25 motifs predicted, Cluster I contained 18 motifs and Cluster II had 10 conserved motifs, of which three were in common.

### Tandem and segmental genome duplications of OsCESA/CSL

**Figure 2:**
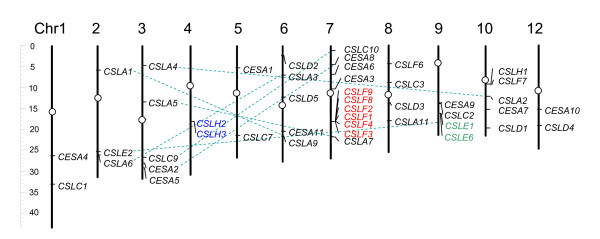
**Chromosomal distribution, and tandem and segmental genome duplications of the *OsCESA*/*CSL *gene family**. The scale on the left is in megabases (Mb). The ovals on the chromosomes (vertical bars) indicate the positions of centromeres; the chromosome numbers are shown on the top of each bar. The segmental duplication genes are connected by a straight broken line, and the tandem duplicated genes are colored.

The *OsCESA*/*CSL *members are distributed on 12 chromosomes of rice (Figure 2). As reported by Burton et al. (2006) [20], members of the *OsCLSF *(*9*, *8*, *2*, *1*, *4*, &*3*) are physically linked within a region of approximately 118 kb of rice chromosome 7. We discovered two additional tandem duplication sets (*OsCSLH2*/*CSLH3 *and *OsCSLE1*/*CSLE6*) and seven segmental duplication sets (*OsCESA2*/*CESA8, OsCSLA1*/*CSLA9, OsCSLA2*/*CSLA4, OsCSLA5*/*CSLA7, OsCSLA6*/*CSLA3, OsCSLC9*/*CSLC10 *and *OsCSLE2*/*CSLE6*) that were assigned to the TIGR segmental duplication blocks at a maximal length distance permitted between collinear gene pairs of 100 kb. In most sets, both members (genes) in a segmental duplication set were from same family. The extreme example is from *CSLA *family; eight of nine members in this family are in duplicated regions. Moreover, most of the duplicated genes have a relatively close phylogenetic relationship; in particular, in the four sets *OsCESA2*/*CESA8, OsCSLA2*/*CSLA4, OsCSLA5*/*CSLA7*, and *OsCSLC9*/*CSLC10*, two member genes are phylogenetically closest to each other (Figure 1A). Interestingly, the two pairs of segmental sets (*OsCESA2/CESA8 *and *OsCSLC9*/*CSLC10*) join closely in two chromosomes (Figure 2). Of the 45 *OsCESA*/*CSL *genes, 23 are involved in duplication events. Therefore, segmental and large-scale tandem duplication events contributed largely to the expansion of this superfamily. Cluster I families were mainly attributed to tandem duplication, whereas Cluster II likely resulted from segmental genome duplication.

### OsCESA/CSL expressions

**Figure 3:**
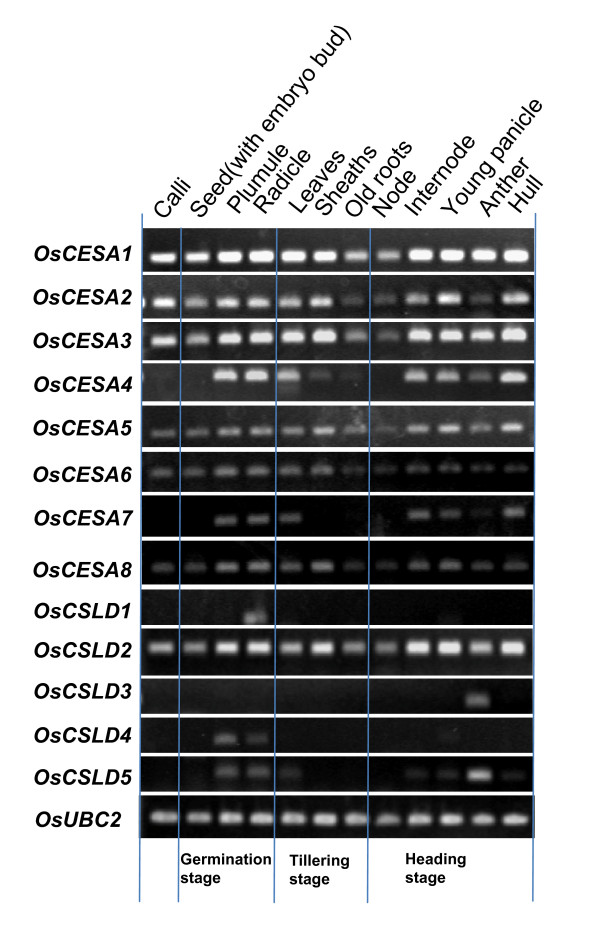
***OsCESA *and *OsCSLD *gene expression patterns by RT-PCR analysis**.

**Figure 4:**
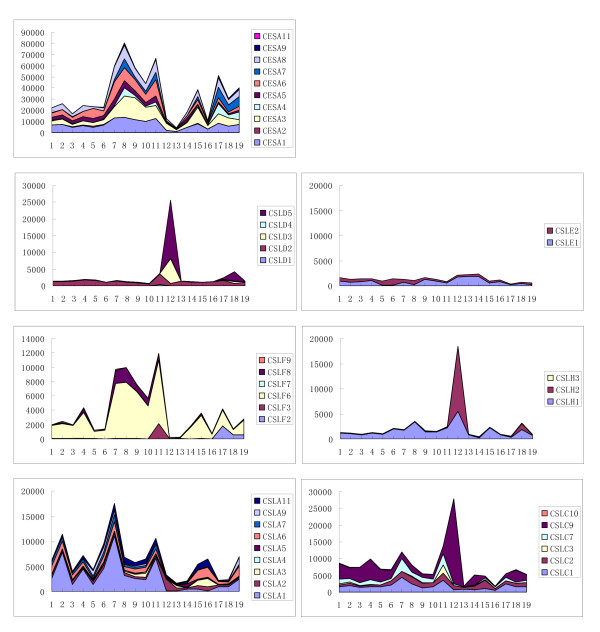
**Accumulative expressions of *OsCESA*/*CSL *genes in representative tissues of rice**. The y-axis indicates the relative expression level of the genes (signal values from the microarray data) and it is arbitrary. The x-axis indicates the tissues across development stages with 1-3: Calli; 4: Seed imbibition; 5: Young panicle stages 3-5; 6: Young panicle; 7: Plumule; 8: Stem; 9: Young leaf and root; 10: Shoot; 11: Radicle and root; 12: Stamen; 13: Flag leaf; 14: Endosperm 1, 2, 3; 15: Sheath; 16: Old Leaf; 17: Hull; 18: Old panicle; 19: Spikelet.

A microarray analysis was conducted for the expression of *OsCESA*/*CSL *genes in two rice varieties (Additional file 2), and the expression patterns of *OsCESA *and *OsCSLD *families were further verified by RT-PCR analysis (Figure 3, Additional file 9). We also demonstrated the expression of *OsCESA*/*CSL *genes in both individual and collective levels (Figure 4). Generally, *OsCESA *genes, with the exception of the Os*CESA11*, exhibited an extensively high expression in most of the tissues examined; in particular, *OsCESA1 *and *OsCESA3 *demonstrated extremely high expression in many tissues over different developmental stages of the life cycle (Figures 3 and 4). In addition, the accumulative *OsCESA *expression levels were highest in the stem and root, but were relatively low in the flag leaf and stamen (Figure 4). Of the *OsCSL *families, six *OsCSL *members (*CSLA1*, *CSLC9*, *CSLD2*, *CSLE1*, C*SLF6 *and *CSLH1*) were expressed in all of the tissues examined. In contrast, other *OsCSL *genes showed tissue-specific expression. For instance, *CSLD3 *& -*5, CSLH2 *and *CSLC*9 showed high stamen-specific expression, whereas *CSLA5, CSLD1 *and *CSLD4 *were specific in the endosperm, radicle and plumule, respectively. The accumulative expression of all the *CSL *genes in a family is also depicted in Figure 4. The overall expression of the family of *CSLD *genes is highest in the stamen and lowest in the shoot of seedlings with two tillers. The total expression of the *CSLA *genes was highest in plumules (mostly contributed by *CSLA1 and 6*) and was followed by high expression in radicles (roots) and calli, with the lowest expression detected in flag leaves. The total expression of *CSLC *was higher in the stamen and plumule/radicles, but was lower in leaves. Collectively the expression of the genes of the whole family often accumulated to high levels in one or more of the tissues for which the *CSL *members showed preferences. This may indicate functional homoplasy among the members in a family although most of them exhibit different expression patterns.

### Expression divergence of OsCESA/CSL genes in duplication

**Figure 5:**
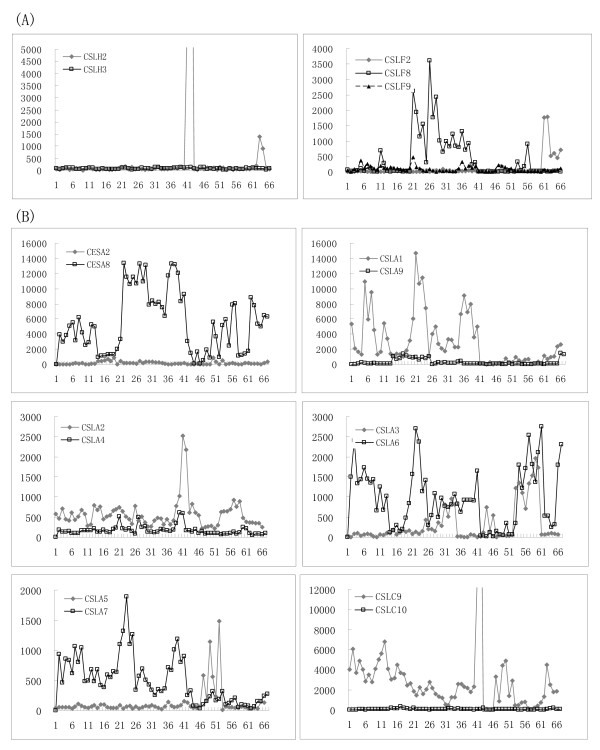
**Expression patterns of the *CESA*/*CSL *genes as tandem duplicates (A) and segmental duplicates (B) in rice**. The x-axis represents the developmental stages as given in Additional file 1. The y-axis represents the raw expression values obtained from the microarray analysis.

We further observed the expression profiling of the duplicated *OsCESA *and *OsCSL *genes. The expression of the two duplication sets *OsCSLE1*/*OsCSLE6 *and *OsCSLE2*/*OsCSLE6 *were not included in the analysis because we lacked the corresponding probe set of *OsCSLE6*. The expression profile of the eight remaining sets of *OsCESA*/*CSL *genes (two tandem duplication sets and six segmental duplication sets) with the corresponding probes was analyzed. We found a divergent expression pattern within a duplicated set (Figure 5). The pairwise expression correlation coefficients (r values) of the duplicated *OsCESA*/*CSL *genes were below the level of significance at P = 0.05 (data not shown). Of the nine gene sets, only *CSLA2 *and *CSLA4 *in a segmental duplication set (*CSLA2*/*CSLA4*) exhibited a relatively similar expression pattern. The fate of four pairs (*CSLH2*/*CSLH3*, *CESA2*/*CESA8*, and *CSLC9*/*CSLC10*) could be described as nonfunctionalization, where one member of the set lost expression in all tissues, while the other showed strong expression. In the other duplication sets, the expression patterns of both member genes were partial complementary and/or overlapped. Comparison of expression pattern shifts of the duplicated genes of the *OsCESA*/*CSL *superfamily could reflect the divergence hypotheses that a duplicate gene pair might be involved in: nonfunctionalization, subfunctionalization and neofunctionalization [40].

### OsCESA/CSL co-expression profiling

**Figure 6:**
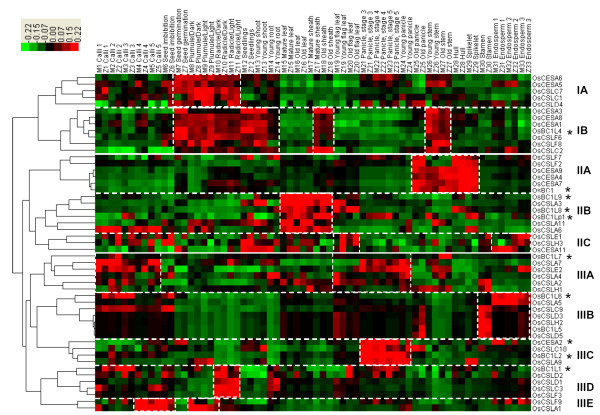
***OsCESA*/*CSL *co-expression profiling in rice**. The color scale representing the relative signal values is shown above (green refers to low expression; black refers to medium expression and red refers to high expression). Genes of the *brittle culm 1 like *family (*OsBC1L*) were marked with asterisks.

Because many genes of COBRA-like proteins, including the *brittle culm1 like *family (*OsBC1L*), have been investigated for cell wall biosynthesis in *Arabidopsis *and rice [41-44], the *OsBC1L *genes were referred as markers of *OsCESA*/*CSL *co-expression patterns in this study. Based on the hierarchical cluster analysis, the *OsCESA*/*CSL *family can be classified into three major groups with ten distinct groups that exhibit a complementary expression pattern spanning 33 tissues from entire life cycle of two rice varieties (Figure 6). Each group consists of multiple *OsCESA*/*CSL *members, which show predominant co-expression in tissues with distinct cell wall constitutions (Table 2).

**Table 2:** Cell wall composition (%) of seven representative tissues in rice

Tissues	Cellulose	Hemicelluloses			Pectins			
			
		Hexose	Pentose	Total	Hexose	Pentose	UroA	Total
Calli	23.8 (4.2)*	35.1	64.9	65.4 (11.5)	23.0	23.9	53.0	10.8 (1.9)
Seedling leaves	48.8 (15.7)	31.1	68.9	44.8 (14.4)	33.1	26.5	40.4	6.4 (2.1)
Seedling roots	54.0 (20.5)	35.1	64.9	42.5 (16.1)	45.3	30.9	23.8	3.5 (1.3)
Young stem	33.8 (11.1)	64.0	36.0	63.5 (20.9)	34.5	27.5	38.0	2.7 (0.9)
Old stem	38.3 (20.6)	67.3	32.7	60.1 (32.3)	30.3	21.1	48.5	1.7 (0.9)
Hull	56.4 (26.6)	22.7	77.3	41.1 (19.4)	36.1	30.1	33.8	2.5 (1.2)
Stamen	29.7 (2.3)	24.9	75.1	29.0 (2.3)	34.3	30.0	35.7	41.3 (3.3)

* % of wall polysaccharide based on the tissue dry weight; the absolute values are bracketed.

Generally, Group IA showed high co-expression in the young vegetative tissues (M7/Z7-M11/Z11) typical of the primary cell wall, and Group IB exhibited additional co-expression in other vegetative tissues (e.g., seedlings, young shoots and stems). Five *OsCESAs *(*5, -6 *and *1, -3, -8*) were strongly co-expressed in those two groups, suggesting that OsCESA1, -3 & -8 may form a cellulose synthase complex for primary cell wall biosynthesis. However, while *OsCESA1 *and *OsCESA8 *are tightly co-expressed, there are some differences in expression between *OsCESA3 *and *OsCESA1 *&*-8 *(Figure 6). We observed that *OsCESA*3 had exceptionally low expression in the plumule and radicle (M8/Z8-M11/Z11), where the expression of *OsCESA5*/*OsCESA6 *is relatively high (Figure 6). This observation might indicate the partial complementation of *OsCESA3 *by *OsCESA5 *& -*6 *in the expression pattern. In comparison to Group I, Group II showed co-expression in three tissues rich in secondary cell walls (old panicle, hull and spikelet) (Figure 6). However, three *OsCESAs *(C*ESA4, -7 & -9*) in the group also showed a co-expression pattern that overlapped with Group IB in young and old stem tissues, which represent the transition stage from primary to secondary cell wall synthesis. Thus, OsCESA4, -7 & -9 may be organized as a cellulose synthase complex involved in secondary cell wall synthesis. In contrast, Group III appeared to show co-expression in diverse tissues harboring specific cell wall structures. For instance, five *OsCSL *genes of Group IIIB demonstrated high co-expression in the stamen (M31/Z31), a tissue that contains extremely high levels of pectins (Table 2), and Group IIIC showed co-expressions in four early stages of panicle development. Co-expression was detected between the *OsCESA *and *OsCSL *families in all ten groups; we also observed strong co-expression between the *OsCESA*/*CSL *and *OsBC1L *families in seven groups, each containing at least one *OsBC1L *family gene. For instance, *OsBC1 *and *OsBC1L5 *both have correlation coefficients (r values) above 0.94 with respect to their relevant *OsCESA*/*CSL *genes. Interestingly, this extensive co-expression was only found between *BC1L *and *OsCESA*/*CSL*. There are no such extensive relationships found between *OsCESA*/*CSL *with other gene families, such as cellulase (including Korrigan), lignins and expansins (data not shown).

### Comparative co-expression analyses with Arabidopsis

**Figure 7:**
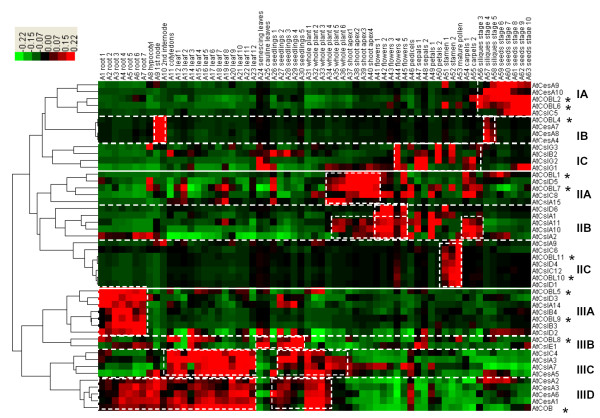
***AtCESA*/*CSL *gene co-expression profiling in *Arabidopsis***. The color scale representing the relative signal values is shown above (green refers to low expression; black refers to medium expression and red refers to high expression). Genes of the *COBRA *like family were marked with asterisks.

Using the *Arabidopsis *public database, we presented a co-expression profiling of 63 tissue samples, and compared it with rice (Figure 7, Table 3). Based on hierarchical clustering, the expression pattern of the *AtCESA*/*AtCSL *genes could also be divided into three major groups (Figure 7). In contrast, the expression patterns of the *CESA*/*CSL *genes in both species are summarized in Table 3. Clearly, the expression patterns of the genes of the *AtCESA*/*AtCSL *superfamily fell into groups similar to those of the *OsCESA*/*CSL *genes. As an example of genes showing a similar expression pattern, *AtCESA1, -3 & -6 *showed high co-expression in the tissues of the primary cell wall, whereas *AtCESA4, -7 & -8 *were co-expressed in the secondary cell wall tissues. As an example of genes showing a different expression pattern, there was no *AtCESA *gene, like *OsCESA3*, showing an exceptionally low expression level. In addition, distinct *CSL *co-expressions were compared between rice and *Arabidopsis *(Table 3). For example, a group of IC genes (*AtCSLG1*, *-2*, &*-3 *and *AtCSLB2*) was specifically expressed in flower organs (carpels or sepals) in *Arabidopsis*, while the *OsCSLF *genes (*OsCSLF2 *&*-7*) were preferentially expressed in the hull of rice. Thus, the gene expression pattern may reflect both the similarities and differences in the cell wall composition of rice and *Arabidopsis*.

**Table 3:** Comparison of CESA/CSL co-expression in rice and Arabidopsis

	Rice			*Arabidopsis*	
	
Groups	Tissues	Genes	Groups	Tissues	Genes
***Preferential expression in young vegetative tissues***					
IA	Youngest seedling (w/o root)	*CESA5,6; CSLC1,7; CSLD4*	IIIC	Youngest seedling (w/o root)	*CESA5; CSLC4; CSLA3,7*
IB	Young seedling (w/root)	*CESA1,3,8; CSLF6,8; CSLC2; BC1L14*	IIID	Young seedling (w/root)	*CESA1,3,6,2; COB*
***Preferential expression in reproductive stages***					
/	/	/	IA	Seed, silique	*CESA9,10; COBL2,6*
IIA(a)	Hull	*CSLF2, 7*		Silique	*CSLC5*
IIA(b)	Stem, hull	*CESA4,7,9; BC1*	IB	Stem, silique	*CESA4,7,8;COBL4*
/	/	/	IC	Flowers(sepals)	*CSLG2,3; CSLB2*
/	/	/		Flowers(Carpels)	*CSLG1*
IIB	Flag leaf and sheath	*CSLE1; CSLH3; CESA11*	/	/	/
IIC	Flag leaf and endosperm	*CSLA3,6,11; BC1L9*	/	/	/
***Preferential expression in tissues undergoing rapid extension***					
/	/	/	IIA	Shoot apex, Cauline leaf, Carpel	*CSLD5; CSLC8; CSLA15; COBL1,7*
/	/	/	IIB	Flowers (Carpels)	*CSLD6; CSLA1,2,10,11*
IIIB	Stamen and endosperm	*CSLC9,CSLD3,5; CSLH2; BC1L5*	IIC	Stamen (Pollen)	*CSLA9; CSLC6,12; CSLD1,4; COBL10,11*
IIID	Radicle and root	*CSLD1,2; CSLC3; CSLF3; BC1L1*	IIIA, IIIB	Roots	*CSLD2,3; CSLA14; CSLB3,4;COBL5,8,9; CSLE1*
IIIA	Callus and young panicle	*CSLA2,4,7; CSLE2; CSLH1*	/	/	/
IIIC	Young panicle	*CESA2,9; CSLC10; BC1L2*	/	/	/
IIIE	Seed imbibition	*CESA1; CSLF9*	-	-	-

"/" indicates no corresponding tissues or the unavailability of data

## Discussion

The previous characterization of the rice *OsCESA*/*CSL *family was focused on phylogenetic and gene structure analyses [12,28]. Hazen et al. (2002) identified 37 *OsCSL *genes [28]; however, some of the *CSL *genes are pseudogenes, and these have now been updated http://waltonlab.prl.msu.edu/CSL_updates.htm. For examples, *CSLC4*, *-5*, *-6 *&*-8 *were verified as pseudogenes and were not included in this study. The *OsCSLA8 *(LOC_Os09g39920.1) gene was recently annotated as a retrotransposon in TIGR version 6.1, while *OsCSLA10 *(DAA01745.1) identified in the NCBI database was actually the same as *OsCSLA4 *and now has been excluded. These updated *OsCESA*/*CSL *genes were indentified and characterized in this study. We performed expression, co-expression and comparative co-expression analyses of this superfamily. The results, coupled with the bioinformatic analysis of phylogeny, gene structure, motif constitution, genome organization and gene duplication, could provide an innovative approach and important clues toward understanding the roles of the *CESA*/*CSL *superfamily in cell wall biosynthesis in higher plants.

### CESA/CSL evolution and classification

In principle, gene families are extended by three major mechanisms: segmental duplication, tandem duplication and retroposition [45,46]. Here we confirmed that both tandem and segmental duplication events were largely responsible for the expansion of the *OsCESA*/*CSL *family. Interestingly, we characterized two clusters of *OsCESA*/*CSL *and concluded that they not only differ in phylogeny and motif constitution, but that they also expanded in the following distinct ways: Cluster I (*OsCESA*/*CSLD*, *E*, *F *and *H*) arose mainly from the tandem duplication, and Cluster II (*CSLA*/*CSLC*) resulted from the segmental duplication. These results support a previous report claiming that *CSLA*/*CSLC *has a different evolutionary origin compared to other *CSL *families [12]. In terms of the duplicated gene expression, we observed that two genes in a duplication set show a strongly contrasting expression pattern. The fate of duplicated genes in *OsCESA*/*CSL *could be described as nonfunctionalization, subfunctionalization and neofunctionalization. None of the genes in a segmental duplication set have similar expression patterns. The latter findings are consistent with a previous report whereby growth-related genes were sensitive to high dosage of gene expressions, and stress responsive genes were tolerant to high dosage [47].

The comparison of the *CESA *expression patterns among seven plant species (rice, barley, maize, poplar, cotton, eucalyptus and *Arabidopsis*) is depicted in the unrooted neighbor-joining tree (Additional file 10). Most clusters contain genes from both monocot and dicot plants, and most orthologs show a higher similarity than paralogs in the *CESA *family, indicating that some gene expansion may have arisen earlier than when the divergence(s) of the species occurred. The latter result is supported by reports whereby the orthologous genes in a cluster show a similar expression pattern in primary and secondary cell walls [48,49]. Furthermore, we compared the expression patterns of some *CSL *homologs in *Arabidopsis*, rice, barley and other species, and a striking similarity was observed in the close orthologous genes across species (Additional file 11). We also observed similarities of *CSL *orthologs in other aspects such as gene duplication and intron-exon structure (data not shown). Thus, such observations could be helpful in the prediction of gene expression patterns of orthologs in cereal species and other higher plants.

### Analysis of OsCESA functions

Patterns of co-expression can reveal networks of functionally related genes and provide a deeper understanding of the processes required to produce multiple gene products [50]. The genome-wide expression analysis of the *CESA *family could provide insights into the potential functions of its members in cell wall biosynthesis. Almost all *OsCESA *genes are highly expressed in the tissues we examined, confirming their major roles in the biosynthesis of cellulose, the main component of plant cell walls. The co-expression profiling of the *CESA *genes can somehow indicate their protein interaction/association as an essential synthase complex for cellulose biosynthesis. Despite the use of the mutant analysis and co-immunoprecipitation in *Arabidopsis *[3,5,51], the application of these approaches in the identification of the CESA complex in other higher plants, such as rice, maize and barley has not been reported.

**Figure 8:**
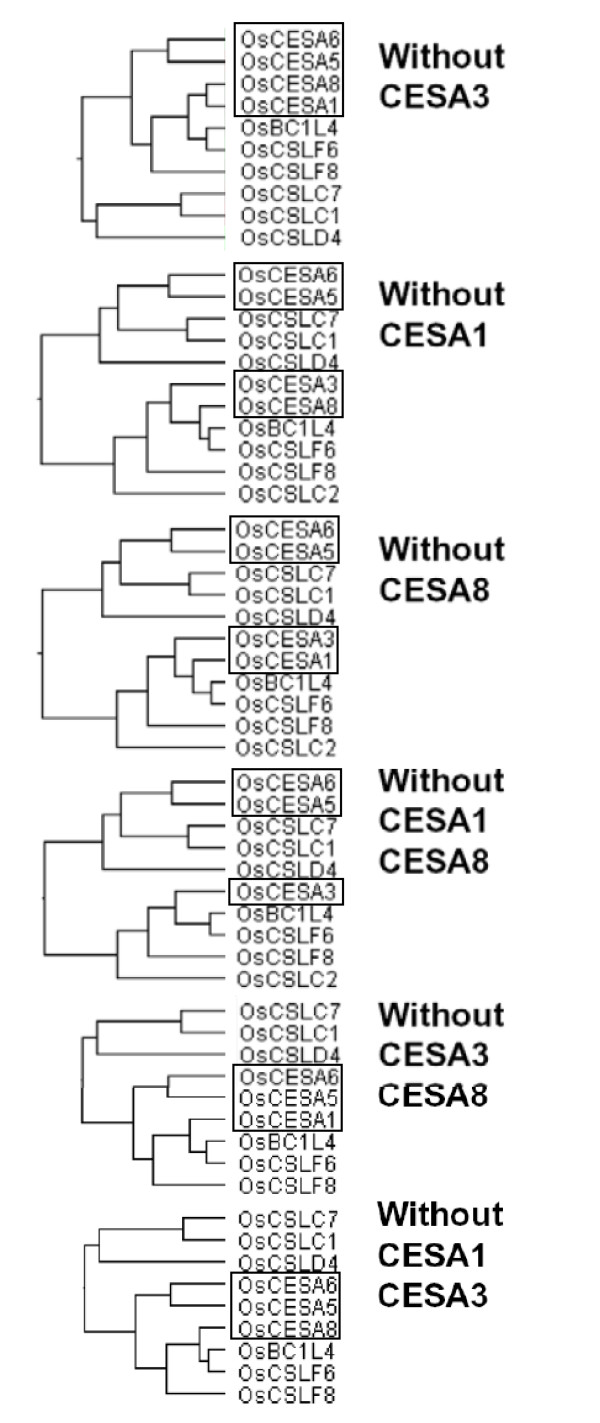
**Gene co-expression profiling of *OsCESA *by "Artificial-mutant" analysis in all the tissues examined**.

**Figure 9:**
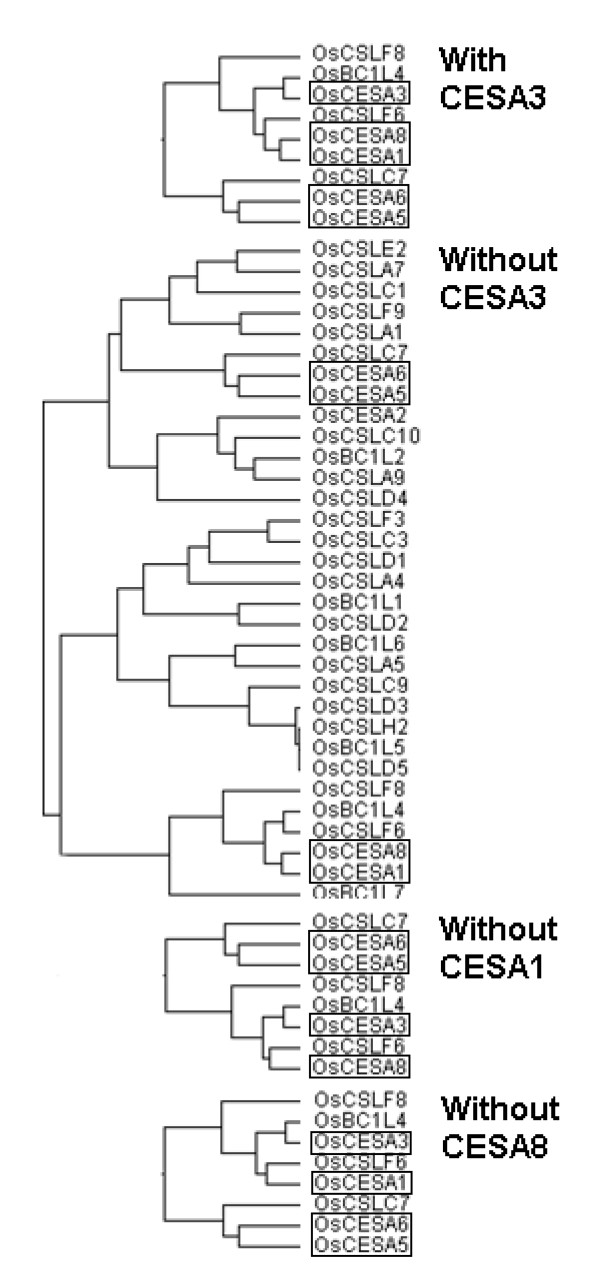
**Gene co-expression profiling of *OsCESA *by "Artificial-mutant" analysis; data from the plumule and radicle tissues were excluded**.

In this work, therefore, we utilized an alternative approach via the integrative analysis of gene co-expression profiling and developmental regulations. First, we confirmed the formation of two distinct cellulose synthase complexes, AtCESA1, -3, & -6 and AtCESA4, -7, & -8, in *Arabidopsis *from our AtCESA co-expression profiling data (Figure 7). Similarly, we can assume that OsCESA1, -3 & -8 and OsCESA4, -7 & -9 may be two synthase complexes involved in primary and secondary cell wall synthesis in rice, respectively (Figure 6, Table 2), which provides clues on the physical interactions of proteins in the synthase complexes. The co-expression profiling in *Arabidopsis *in this study, however, could not further verify the previous finding of *AtCESA6 *as partial redundant gene with *AtCESA2 & -5 *[4,5], probably because of the lack of essential expression data of *Arabidopsis *tissues from the public microarray data (Figure 7). Similarly, we could assume *OsCESA3 *to be a partially redundant candidate gene with *OsCESA5*/*OsCESA6 *given its low transcript level in specific tissues (plumule and radicle), where the expression of *OsCESA5*/*OsCESA6 *is relatively high (Figure 6). In other words, *OsCESA5 *or *-6 *may be partially redundant with *OsCESA3 *in those specific tissues. Eventually, we demonstrated the partial redundancy of *OsCESA5 *or -6 with OsCESA3 by a novel approach, the "artificial-mutant" analysis of gene co-expression profiling (Figures 8 and 9, Additional file 12 and 13). While *OsCESA3 *was artificially deleted, the hierarchical cluster analysis showed that *OsCESA1 *&*-8 *clustered together with the *OsCESA5 *and *OsCESA6*. This result might indicate that OsCESA1 & -8 form a synthase complex with OsCESA5 or OsCESA6 (Figure 8). However, deleting either *OsCESA1 *or *OsCESA8 *did not disrupt the above organization (Figure 8). Even after the double deletion of *OsCESA3*/*OsCESA1 *or *OsCESA3*/*OsCESA8*, OsCESA5 and OsCESA6 could somehow still organize a complex with either OsCESA1 or OsCESA8 (Figure 8). Clearly, the data are in support of our assumption. When the gene expression data in the plumule and radicle tissues were not included in the hierarchical cluster analysis, *OsCESA1 *&*-8 *could not form a group with *OsCESA5 *or *OsCESA6 *when *OsCESA3 *was artificially deleted (Figure 9). Thus, we believe that partial redundancy occurs in the specific development stages/tissues (such as plumule and radicle) of rice.

### Characterization of the OsCSL family

Several *OsCSL *genes were demonstrated to exhibit relatively tissue-specific expression, indicating their specific/unique roles for wall polysaccharides synthesis or their potentially functional complements for appropriate cell wall synthesis. For instance, in the pectin-rich and cellulose-less stamen tissue (Table 2), all *OsCESAs *have a relatively low transcript level, but three *OsCSLs *(*OsCSLC9, OsCSLD5 *and *OsCslH2*) exhibit specifically high expression. In addition, all six *OsCSL *families appear to have at least one highly expressed gene (*CSLA1*, *CSLC9*, *CSLD2*, *CSLE1*, C*SLF6 *and *CSLH1*) in all the tissues we examined, therefore suggesting that the entire *OsCSL *family is essential for cell wall biosynthesis.

The analysis of co-expression profiling and developmental regulations, together with a comparison with *Arabidopsis*, can be used for the characterization of *OsCSLs*. As described above, we concluded that ten co-expressed groups are expressed in cells/tissues with different cell wall constitution. Based on this information, we could find clues about the predominant roles of *OsCSL *genes in cell wall biosynthesis. For example, *OsCSLF2 *and *OsCSLF7 *in Group IIA may have quite a different role from other *OsCSLF *genes in Groups IB, IIID an IIIE (Figure 6). *OsCSLF2 *and *OsCSLF7 *show a uniquely high co-expression pattern with *OsCESA4, -7 & -9 *in the hull/spikelet tissue typical of secondary cell walls (Figure 6); however, they both have a much lower transcript level than *OsCSLF6 *and *OsCSLF8 *(Figure 4). Because there are pentose-rich hemicelluloses in the hull tissue (Table 2), we assume that OsCSLF2 and OsCSLF7 may also encode other synthase enzymes besides the β-(1,3-1,4)-glucan synthase that was previously characterized. In addition, comparison of co-expression profiling in the stamen tissue between rice (Group IIIB) and *Arabidopsis *(Group IIC) suggests that *OsCSLH2 *and *AtCSLA9 *may play a similar or replaceable role in cell wall synthesis (Table 3). We can also infer the functional meanings from the developmental regulations of the gene expression. For an example, the higher expression of *OsCSLD2 *and *OsCSLE1 *was found in older leaves versus young leaves. This result was consistent with the report that *AtCSLD2 *and *AtCSLE1 *apparently exhibit strong increases in expression in old leaves versus young leaves in *Arabidopsis *[25]. The authors proposed that the changes in expression of these two genes may reflect a role in homogalacturonan synthesis, which accumulated to a high level in old leaves. The availability of more detailed information about cell wall composition (e.g., monosaccharide) will help in establishing links between CESA/CSL proteins and the carbohydrates they might synthesize.

## Conclusions

Previous analysis of the functions of *CESA*/*CSL *members on plant cell wall biosynthesis has been focused on biochemical and genetic approaches in the model plant *Arabidopsis*. Here, we performed a validated approach that is applicable in higher plants and successful at finding out useful clues on OsCESA/CSL protein interaction or association. Our approach not only relies on a comprehensive phylogenetic analysis, but it also integrates the characterization of co-expression profiling and regulations, which can reveal very useful clues on the dynamic organization of *OsCESA *proteins as distinct cellulose synthase complexes in primary and secondary cell wall biosynthesis. We also conclude that the co-expression profiling of *OsCESA/OsCSL *and *OsBC1L *can be associated with ten distinct groups in specific cell wall polysaccharide synthesis. In a word, our results provide insights into functional analyses of *CESA*/*CSL *family and of other GT families or cell wall-related genes in rice and other higher plant species.

## Authors' contributions

LW performed all data analyses and drafted the manuscript. KG conducted all data collection and analyses. YT and HH completed chemical tests. YL, BW and XC participated in the growing of the rice and in data interpretation. LP supervised the project and finalized the paper. All authors have read and approved the final manuscript.

## Supplementary Material

Additional file 1 **Tissues and developmental stages throughout the life cycle of two rice varieties**. Click here for file

Additional file 2 **Signal intensities of the probe sets for the *OsCESA*/*CSL *and *OsBC1L *families**. Click here for file

Additional file 3 **Tissues sampled from different developmental stages throughout the life cycle of *Arabidopsis***. Click here for file

Additional file 4 **Signal intensities of the probe sets for the *AtCESA*/*CSL *and *AtCOBL *families**. Click here for file

Additional file 5 **Primers of the *OsCESA*/*CSLD *genes used for RT-PCR analysis**. Click here for file

Additional file 6 **Conserved amino acids in the "D, D, D, QXXRW" motif (depicted in red) of OsCESA/CSL in rice**. Click here for file

Additional file 7 **Motif composition of the OsCESA and CSL protein families**. Click here for file

Additional file 8 **Details of the 25 putative motifs**. Click here for file

Additional file 9 **Expression patterns of the individual genes from *OsCESA *(up) and *OsCslD *(below) families in representative tissues of rice**. The y-axis indicates the relative expression level of the genes (signal values from the microarray data) and it is arbitrary. The x-axis indicates the tissues across development stages with 1-3: Calli; 4: Seed imbibition; 5: Young panicle stages 3-5; 6: Young panicle; 7: Plumule; 8: Stem; 9: Young leaf and root; 10: Shoot; 11: Radicle and root; 12: Stamen; 13: Flag leaf; 14: Endosperm 1, 2, 3; 15: Sheath; 16: Old Leaf; 17: Hull; 18: Old panicle; 19: Spikelet. Click here for file

Additional file 10 **Unrooted phylogenetic tree subjected to the alignment of the deduced amino acid sequences of the OsCESA family genes with full-length CESA protein sequences from other species**. At = *Arabidopsis thaliana*; Eg = *Eucalyptus grandis*; Gh = *Gossypium hirsutum*; Hv = *Hordeum vulgare*; Os = *Oryza sativa*; Ptr = *Populus tremuloides*; and Zm = *Zea mays*. "PCW" and "SCW" indicate primary cell wall and secondary cell wall, respectively. Information about *CESA *refers to At [4,25,48,52], Zm [6], Hv [7], Ptr [8,9], Eg [49]. Click here for file

Additional file 11 **Comparative analysis of the expression patterns of the *CSL *homologs (*CSLD*, *CSLF*, *CSLC *and *CSLA*) in *Arabidopsis*, rice, barley and other species**. Os: rice, At: *Arabidopsis*, Hv: barley, Pt(r): poplar, Na: tobacco; The plus signs indicate the preferential expression, while the minus sign indicates lower expression; The asterisks indicate the genes expressed throughout the tissues examined; The numbers in parentheses indicate the duplicated genes of *OsCESA*/*CSL*; The expression data refer to *AtCESA/CSL *[25,53], *HvCSLF *[54], *HvCSLC *[22], *PtCSLA *[18], *PtrCSLD *and *NaCSLD1 *[55]. Click here for file

Additional file 12 **Gene co-expression profiling of *OsCESA *by "Artificial-mutant" analysis in all the tissues examined**. Click here for file

Additional file 13 **Gene co-expression profiling of *OsCESA *by "Artificial-mutant" analysis; data from the plumule and radicle tissues were excluded**. Click here for file
