# Supplementary material for: Expression profiling and integrative analysis of the CESA/CSL superfamily in rice
Source: BMC Plant Biol. 2010 Dec 20;10:282. doi: 10.1186/1471-2229-10-282 (PMC3022907; doi:10.1186/1471-2229-10-282)
Supplement: Additional file 6 — Conserved amino acids in the "D, D, D, QXXRW" motif (depicted in red) of OsCESA/CSL in rice. [file 1471-2229-10-282-S6.DOC]

**Additional file 6 Conserved amino acids in the “D, D, D, QXXRW” motif (depicted in red) of OsCESA/CSL in rice**

**Q**


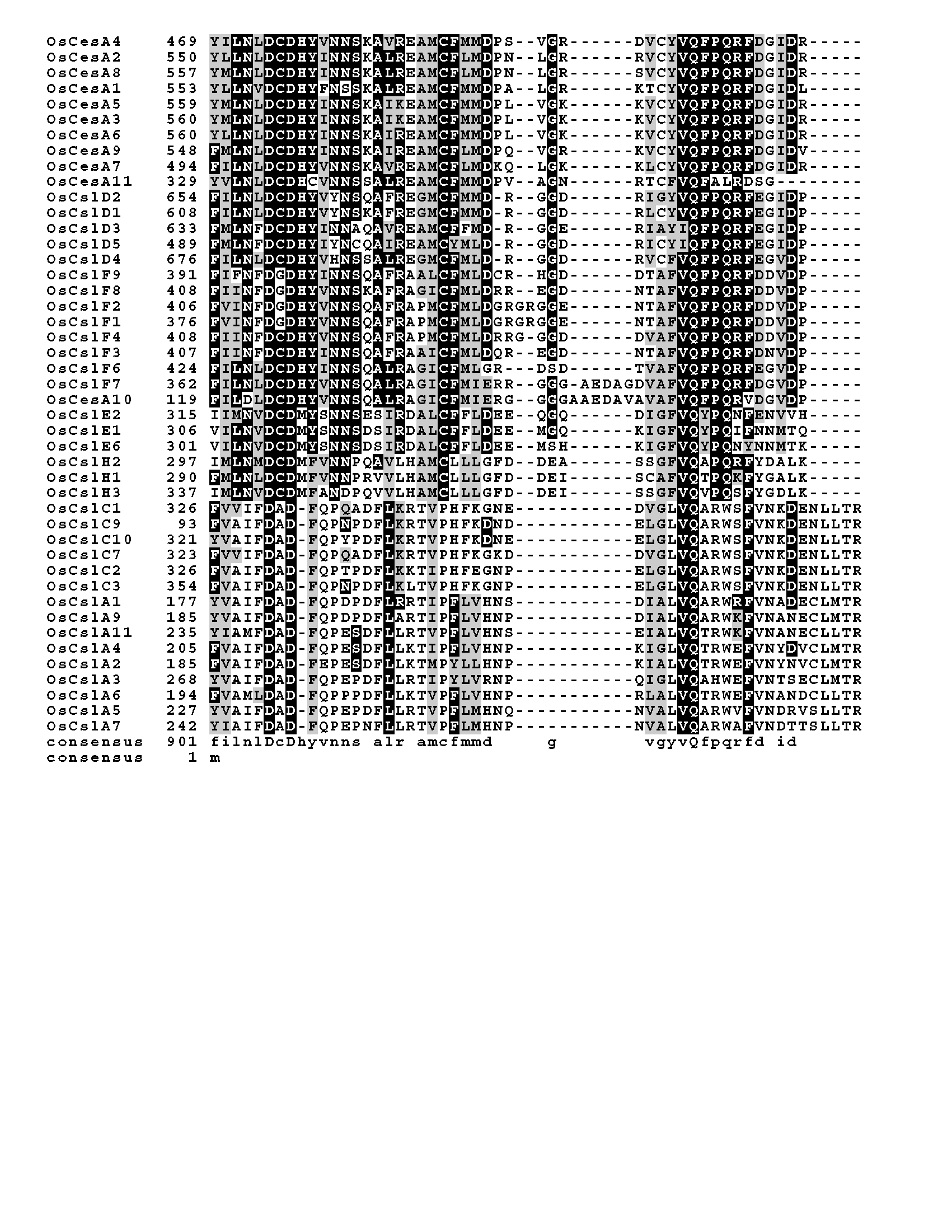


**DXD**


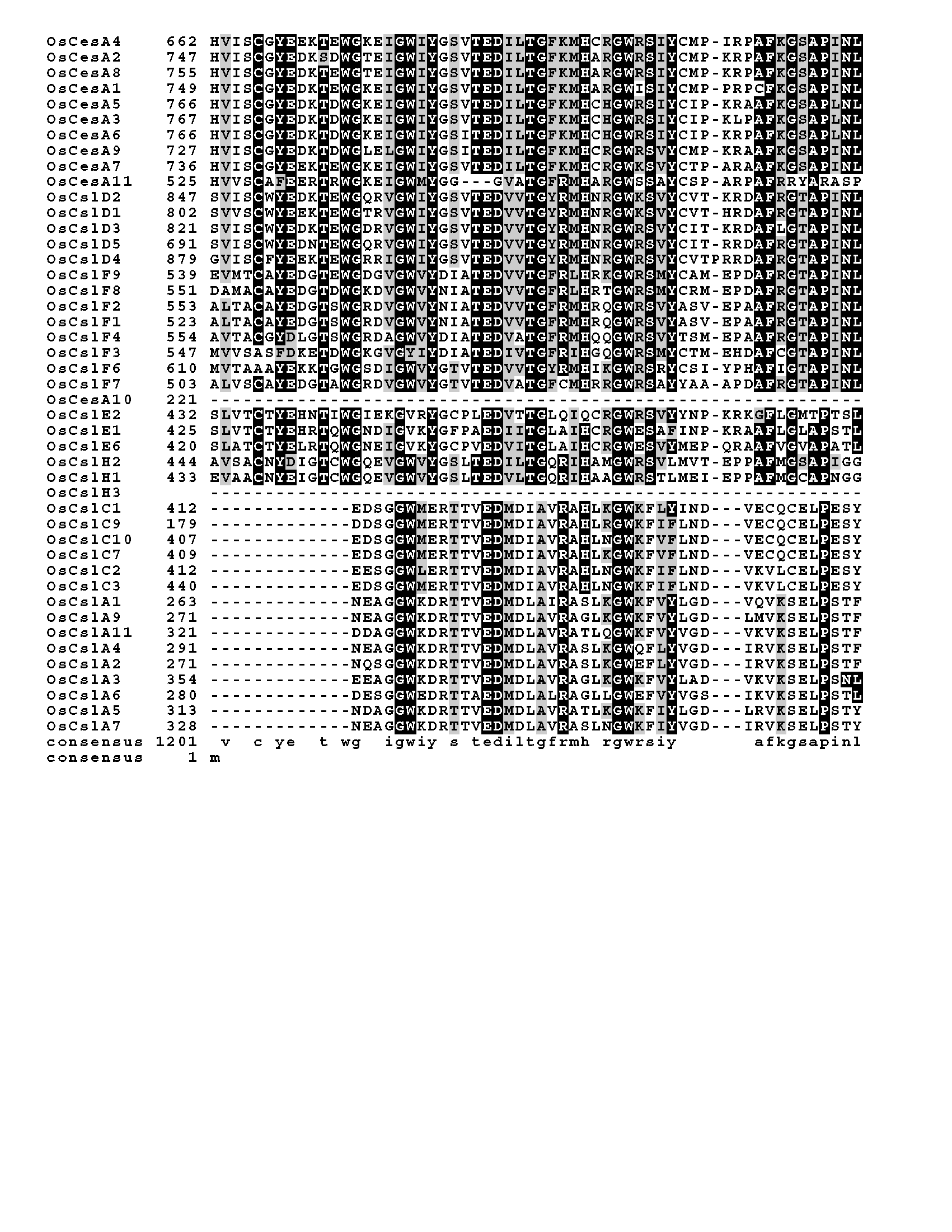


**GW ED GW P**

**QXXRWXXG**


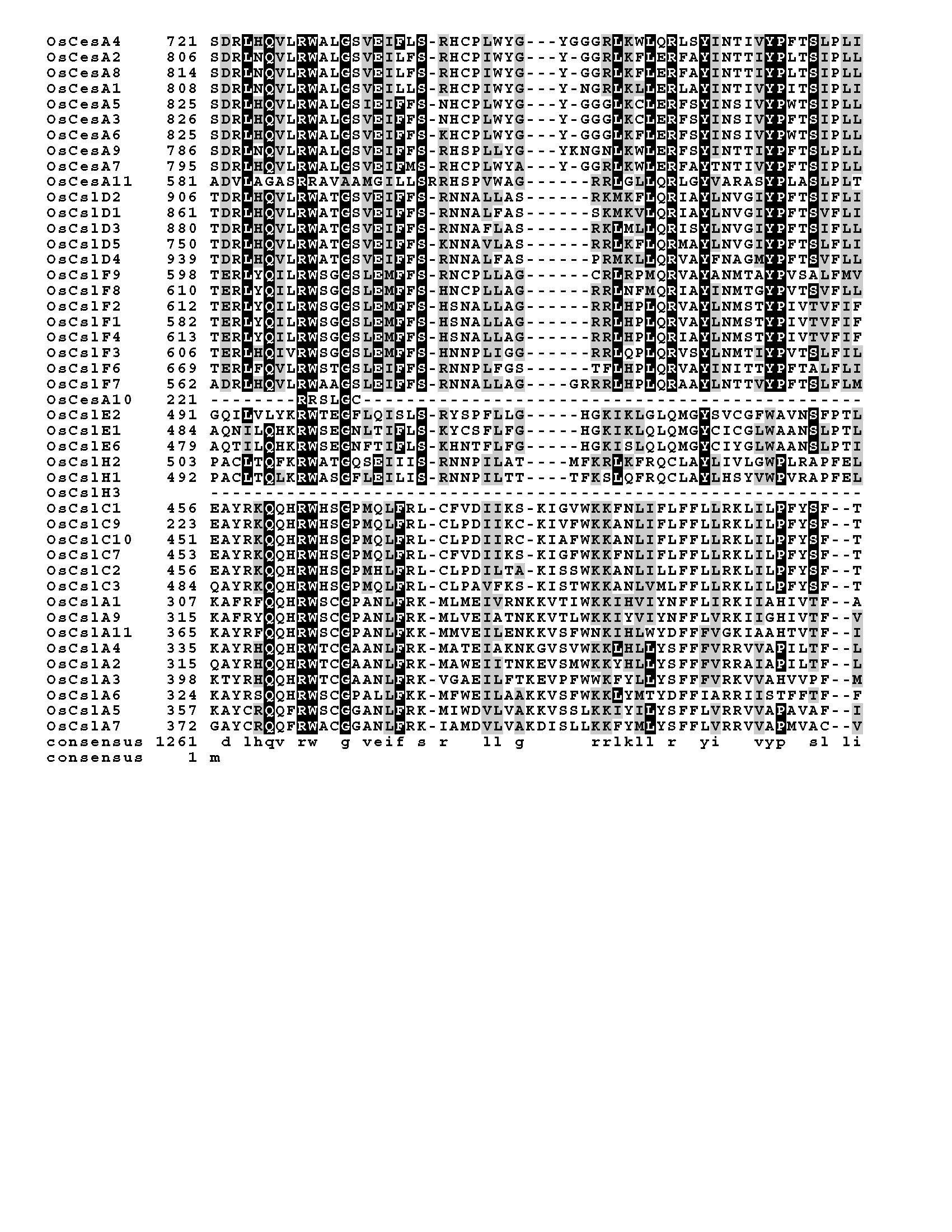


**GW**
