# Supplementary figures and images for: Expression profiling and integrative analysis of the CESA/CSL superfamily in rice
Source: BMC Plant Biol. 2010 Dec 20;10:282. doi: 10.1186/1471-2229-10-282 (PMC3022907; doi:10.1186/1471-2229-10-282)

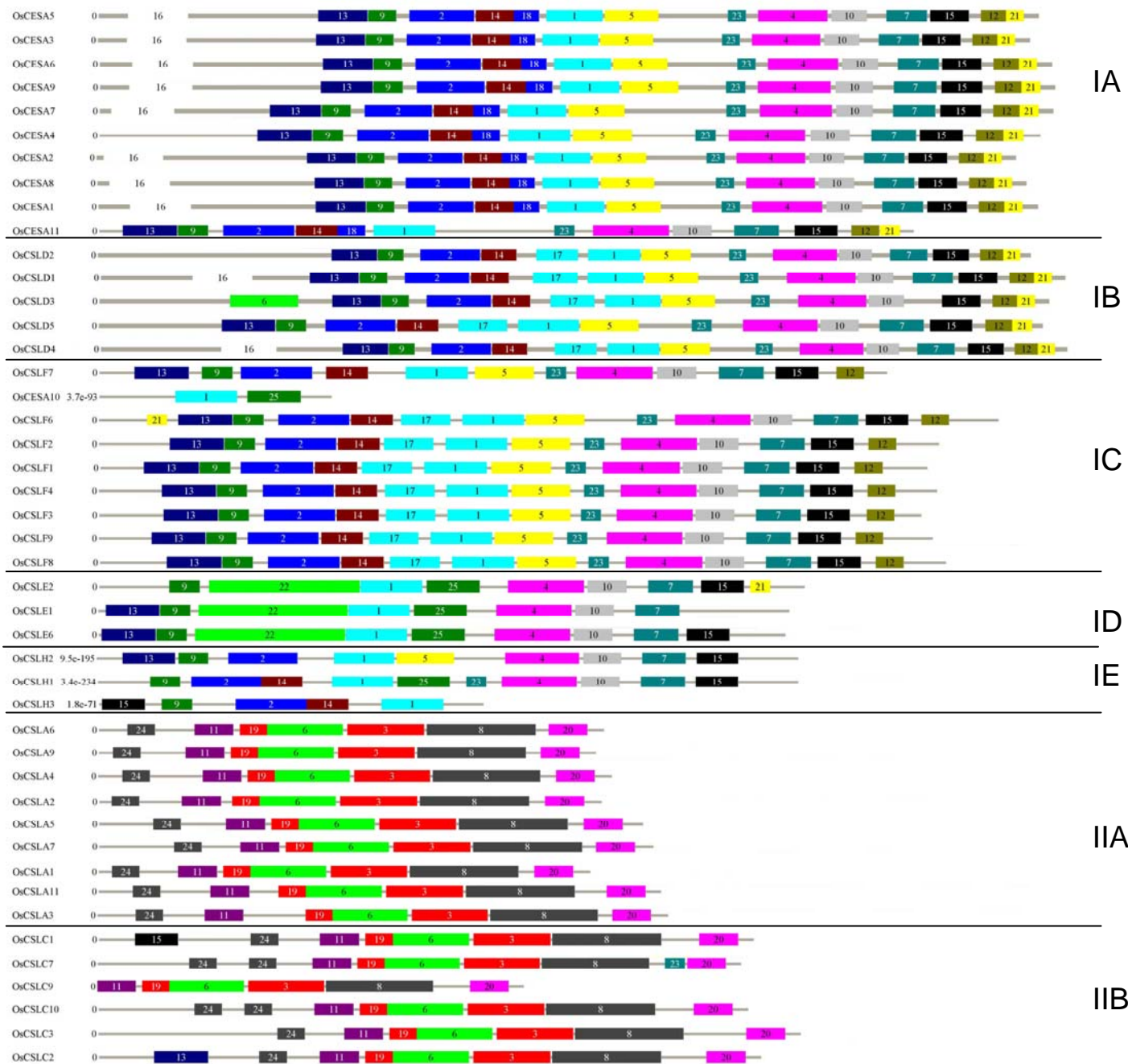

Supplement: Additional file 7 — Motif composition of the OsCESA and CSL protein families. [file 1471-2229-10-282-S7.PDF]
