# Supplementary material for: Expression profiling and integrative analysis of the CESA/CSL superfamily in rice
Source: BMC Plant Biol. 2010 Dec 20;10:282. doi: 10.1186/1471-2229-10-282 (PMC3022907; doi:10.1186/1471-2229-10-282)
Supplement: Additional file 9 — Expression patterns of the individual genes from OsCESA (up) and OsCslD (below) families in representative tissues of rice. The y-axis indicates the relative expression level of the genes (signal values from the microarray data) and it is arbitrary. The x-axis indicates the tissues across development stages with 1-3: Calli; 4: Seed imbibition; 5: Young panicle stages 3-5; 6: Young panicle; 7: Plumule; 8: Stem; 9: Young leaf and root; 10: Shoot; 11: Radicle and root; 12: Stamen; 13: Flag leaf; 14: Endosperm 1, 2, 3; 15: Sheath; 16: Old Leaf; 17: Hull; 18: Old panicle; 19: Spikelet. [file 1471-2229-10-282-S9.DOC]

**Additional file 9 Expression of the individual genes from *OsCESA* (up) and *OsCslD* (below) families in representative tissues of rice**

The y-axis indicates the relative expression level of the genes (signal values from the microarray data) and it is arbitrary. The x-axis indicates the tissues across development stages with 1-3: Calli; 4: Seed imbibition; 5: Young panicle stages 3-5; 6: Young panicle; 7: Plumule; 8: Stem; 9: Young leaf and root; 10: Shoot; 11: Radicle and root; 12: Stamen; 13: Flag leaf; 14: Endosperm 1, 2, 3; 15: Sheath; 16: Old Leaf; 17: Hull; 18: Old panicle; 19: Spikelet
