# Supplementary material for: Expression profiling and integrative analysis of the CESA/CSL superfamily in rice
Source: BMC Plant Biol. 2010 Dec 20;10:282. doi: 10.1186/1471-2229-10-282 (PMC3022907; doi:10.1186/1471-2229-10-282)
Supplement: Additional file 10 — Unrooted phylogenetic tree subjected to the alignment of the deduced amino acid sequences of the OsCESA family genes with full-length CESA protein sequences from other species. At = Arabidopsis thaliana; Eg = Eucalyptus grandis; Gh = Gossypium hirsutum; Hv = Hordeum vulgare; Os = Oryza sativa; Ptr = Populus tremuloides; and Zm = Zea mays. "PCW" and "SCW" indicate primary cell wall and secondary cell wall, respectively. Information about CESA refers to At [4,25,48,52], Zm [6], Hv [7], Ptr [8,9], Eg [49]. [file 1471-2229-10-282-S10.DOC]

**Additional file 10 Unrooted phylogenetic tree subjected to the alignment of the deduced amino acid sequences of the OsCESA family genes with full-length CESA protein sequences from other species**

At = *Arabidopsis thaliana*; Eg = *Eucalyptus grandis*; Gh = *Gossypium hirsutum*; Hv = *Hordeum vulgare*; Os = *Oryza sativa*; Ptr = *Populus tremuloides*; and Zm = *Zea mays*. “PCW” and “SCW” indicate primary cell wall and secondary cell wall, respectively. Information about *CESA* refers to At [4, 25, 48, 52], Zm [6], Hv [7], Ptr [8, 9], Eg [49]
