# Supplementary material for: Expression profiling and integrative analysis of the CESA/CSL superfamily in rice
Source: BMC Plant Biol. 2010 Dec 20;10:282. doi: 10.1186/1471-2229-10-282 (PMC3022907; doi:10.1186/1471-2229-10-282)
Supplement: Additional file 11 — Comparative analysis of the expression patterns of the CSL homologs (CSLD, CSLF, CSLC and CSLA) in Arabidopsis, rice, barley and other species. Os: rice, At: Arabidopsis, Hv: barley, Pt(r): poplar, Na: tobacco; The plus signs indicate the preferential expression, while the minus sign indicates lower expression; The asterisks indicate the genes expressed throughout the tissues examined; The numbers in parentheses indicate the duplicated genes of OsCESA/CSL; The expression data refer to AtCESA/CSL [25,53], HvCSLF [54], HvCSLC [22], PtCSLA [18], PtrCSLD and NaCSLD1 [55]. [file 1471-2229-10-282-S11.DOC]

**Additional file 11 Comparative analysis of the expression patterns of the *CSL* homologs (*CSLD*, *CSLF*, *CSLC* and *CSLA*) in *Arabidopsis*, rice, barley and other species**

Os: rice, At: *Arabidopsis*, Hv: barley, Pt(r): poplar, Na: tobacco;

The plus signs indicate the preferential expression, while the minus sign indicates lower expression;

The asterisks indicate the genes expressed throughout the tissues examined;

The numbers in parentheses indicate the duplicated genes of *OsCESA*/*CSL*;

The expression data refer to *AtCESA/CSL* [25, 53], *HvCSLF* [54], *HvCSLC* [22], *PtCSLA* [18], *PtrCSLD* and *NaCSLD1* [55]
