# Supplementary material for: Expression profiling and integrative analysis of the CESA/CSL superfamily in rice
Source: BMC Plant Biol. 2010 Dec 20;10:282. doi: 10.1186/1471-2229-10-282 (PMC3022907; doi:10.1186/1471-2229-10-282)
Supplement: Additional file 12 — Gene co-expression profiling of OsCESA by "Artificial-mutant" analysis in all the tissues examined. [file 1471-2229-10-282-S12.DOC]

**Additional file 12 Gene co-expression profiling of *OsCESA* by “Artificial-mutant” analysis in all the tissues examined**
